# Smartphone-Based SPAD Value Estimation for Jujube Leaves Using Machine Learning: A Study on RGB Feature Extraction and Hybrid Modeling

**DOI:** 10.3390/s25082545

**Published:** 2025-04-17

**Authors:** Qi Wang, Ziyan Shi, Kaiyao Hou, Ning Yan, Cuiyun Wu, Xu Li

**Affiliations:** 1 College of Information Engineering, Tarim University, Alaer 843300, China; 15703411873@163.com (Q.W.); 19590146013@163.com (Z.S.); qvatanen@gmail.com (K.H.); 18295549487@163.com (N.Y.); 2 Key Laboratory of Tarim Oasis Agriculture, Ministry of Education, Tarim University, Alaer 843300, China

**Keywords:** chlorophyll content, jujube leaves, smartphone images, SPAD, precision agriculture

## Abstract

Chlorophyll content in date leaves is critical for fruit quality and yield. Traditional detection methods are usually complex and expensive. This study proposes a rapid detection method for chlorophyll content using smartphone images and machine learning and deep learning models. The SPAD values and RGB images of Xinjiang date palm were collected. The RGB images were preprocessed and their color features were extracted using Python and OpenCV. Through correlation analysis, 21 color features highly correlated with chlorophyll content were selected and downscaled with principal component analysis. Models including SVR, RVM, CNN, CNN-SVR, and CNN-RVM were used for prediction. Among them, the CNN-SVR model showed the most stable performance with R^2^ values of 72.21% and 77.44% on the training and validation sets, respectively, which outperformed the other models. The proposed method is simple, cost-effective, and highly accurate, providing a novel technical approach for accurate management and health monitoring in the date industry. This method has the potential for wide application.

## 1. Introduction

Jujube (*Ziziphus jujuba* Mill.), a nutrient-rich fruit, is widely recognized for its health benefits and economic value, particularly in tropical and subtropical regions of East Asia, North Africa, and the Middle East [1]. China is the leading producer of jujube, with a cultivation history that dates back thousands of years [2]. Over 400 jujube varieties are grown in China, with Xinjiang being the largest production area, accounting for approximately 95% of the global output [3]. The unique geographical and climatic conditions of Xinjiang make it ideal for jujube cultivation, particularly for varieties such as Jun jujube and Hui jujube, which contribute to more than 90% of Xinjiang’s total production [4]. Among these, Jun jujube is highly valued for its rich flesh, sweet-and-sour taste, and abundant nutritional components, including sugars, organic acids, ascorbic acid, and phenolic compounds. These qualities have garnered significant attention in the health food and pharmaceutical industries [5].

The growth quality of jujube trees is closely linked to the chlorophyll content in their leaves [6]. Chlorophyll plays a pivotal role in photosynthesis, directly influencing light energy absorption, nutrient conversion, and plant development. Consequently, it impacts fruit quality parameters such as sugar content and acidity [7]. Moreover, variations in chlorophyll content can reflect the plant’s response to environmental stresses, such as water scarcity or poor soil conditions. Thus, the dynamic monitoring of chlorophyll content holds significant value for agricultural production [8]. By accurately assessing chlorophyll levels, farmers can optimize fertilizer and water usage, thereby enhancing crop yield and fruit quality [9].

Traditional methods for measuring chlorophyll content primarily fall into three categories. The first method involves spectrophotometry, which offers high accuracy and is often regarded as the standard approach. However, it requires specialized laboratory equipment, complex procedures, and high costs, limiting its practical application [10]. The second method utilizes SPAD chlorophyll meters, such as the SPAD-502, which provide non-destructive and rapid measurements by analyzing the optical properties of leaves [11,12]. Despite their advantages, SPAD meters have limitations, including a small measurement area, a susceptibility to leaf thickness and other factors, and high equipment costs, making them less suitable for large-scale agricultural applications [13]. The third method is based on spectral techniques, such as near-infrared spectroscopy [14], chlorophyll fluorescence spectroscopy [15], and multi- or hyperspectral imaging technologies [16]. These methods predict chlorophyll content by analyzing the spectral characteristics of leaves or canopies [17]. While promising results have been achieved in research, these techniques also face challenges due to the high cost of spectral instruments and the complexity of their operation, rendering them impractical for routine large-scale monitoring [18].

With the rapid advancement of smartphone technology and image processing techniques [19,20], image-based SPAD detection methods are emerging as efficient and convenient alternatives. Smartphones, which are widely accessible and equipped with high-quality cameras, can capture RGB images of plant leaves, from which color features can be extracted to estimate chlorophyll content [21]. Compared with traditional spectroscopic and laboratory-based methods, image-based approaches are cost-effective, easy to operate, and do not require expensive instrumentation, making them highly scalable [22]. The core of this method lies in image processing and analysis. Through segmentation, denoising, and other preprocessing steps applied to captured plant images [23], the RGB color features are extracted. These features, combined with the actual chlorophyll content, are used to train predictive models. Incorporating machine learning techniques further enhances prediction accuracy and efficiency.

In recent years, machine learning (ML)- and deep learning (DL)-based image analysis methods have shown remarkable progress in agricultural applications. For instance, convolutional neural networks (CNNs) have been widely adopted for crop disease detection and chlorophyll prediction due to their ability to automatically extract spatial features [24]. For small-sample scenarios, support vector regression (SVR) and relevance vector machines (RVMs) have demonstrated robustness in handling nonlinear relationships through kernel functions [25,26]. Hybrid models (e.g., CNN-SVR) further integrate the strengths of deep feature extraction and traditional regression, improving accuracy while mitigating overfitting risks [27].

This study advances the state-of-the-art technology in three ways. First, while prior research on RGB-based chlorophyll estimation has focused primarily on temperate crops (e.g., soybean, rice) in humid or sub-humid environments, our work targets *Ziziphus jujuba* leaves in the extreme arid agroecology of Xinjiang’s Alar region. The unique environmental challenges here—including intense solar radiation (2556–2991 h of annual sunshine), low precipitation (40–82 mm/year), and high evaporation (1876–2558 mm/year)—induce specific physiological adaptations in jujube leaves, such as altered chlorophyll distribution and cuticle thickness, which render temperate crop models ineffective. Our dataset and modeling framework are thus tailored to this understudied arid-zone context, filling a critical gap in regional precision agriculture.

Second, we introduce an optimized image preprocessing pipeline that leverages the HSV color space for robust background segmentation in controlled lighting. Unlike traditional RGB-only approaches which are prone to illumination bias, our method converts images to HSV to isolate leaf regions using the Hue and Saturation channels, which are less sensitive to brightness fluctuations. This step, combined with median filtering and contour-based ROI extraction, ensures consistent feature extraction even under subtle lighting variations, a key improvement over prior studies that relied on simplistic thresholding or manual segmentation.

Third, while hybrid CNN–regression models have been explored for other crops, our study systematically compares five models—SVR, RVM, CNN, Convolutional Neural Network-Support Vector Regression (CNN-SVR), and Convolutional Neural Network-Relevance Vector Machine (CNN-RVM)—using rigorous five-fold cross-validation and identifies CNN—SVR as the optimal solution for jujube leaves. The superiority of CNN—SVR stems from its integration of CNN’s automatic spatial feature learning and SVR’s robustness to small samples, addressing the overfitting challenges common in deep learning models when applied to limited-size datasets (*n* = 520). This hybrid architecture, validated under the specific physiological and environmental constraints of jujube, offers a novel modeling paradigm for chlorophyll prediction in data-scarce agricultural scenarios.

This study aims to develop a novel method for detecting chlorophyll content in jujube leaves based on smartphone-captured images. Using Python, leaf image color features are extracted and correlated with SPAD values to identify the features most strongly associated with chlorophyll content. Five machine learning and deep learning models—SVR, RVM, CNN, CNN-SVR, and CNN-RVM—are employed to train and predict chlorophyll content. The performance of these models is compared to identify the optimal predictive model. We select SVR, RVM, CNN, and their hybrid variants to validate their synergistic effects on chlorophyll prediction in jujube leaves, aiming to provide a lightweight solution for resource-constrained agricultural settings. Through comparative model analysis, this research seeks to establish an efficient, convenient, and cost-effective tool for chlorophyll content prediction. Such a tool could support precision management and health monitoring in agricultural production, ultimately improving crop yield and quality. Given the widespread accessibility and affordability of smartphones, this method holds great promise for large-scale agricultural management. Its integration into modern agricultural practices could provide a valuable solution for addressing the challenges of real-time crop monitoring and resource optimization.

## 2. Materials and Methods

### 2.1. Study Area Overview

The experiment was conducted at the Horticultural Experimental Station of Tarim University in Alar City, Xinjiang Uygur Autonomous Region (81°29′ E, 40°54′ N). Alar City is located on the northern edge of the Taklamakan Desert, in the upstream region of the Tarim River where the Aksu, Hotan, and Yarkand Rivers converge. The area is characterized by a typical warm temperate extreme continental arid desert climate, with an average annual temperature of 10.7 °C and sparse annual precipitation ranging from 40 to 82 mm. In contrast, the evaporation rate is as high as 1876 to 2558 mm, with abundant sunshine lasting 2556 to 2991 h annually.

The extreme aridity and significant diurnal temperature variations in this region accelerate sugar accumulation in Jun jujube, resulting in superior sweetness and quality. Sufficient sunlight and relatively stable irrigation conditions further ensure healthy growth and high yields. This study was conducted in a 4-year-old jujube orchard with uniformly spaced trees (2 × 1 m rectangular planting density). The selected trees were of the Jun jujube variety.

### 2.2. Data Collection

#### 2.2.1. Leaf Sampling

**Figure 1:**
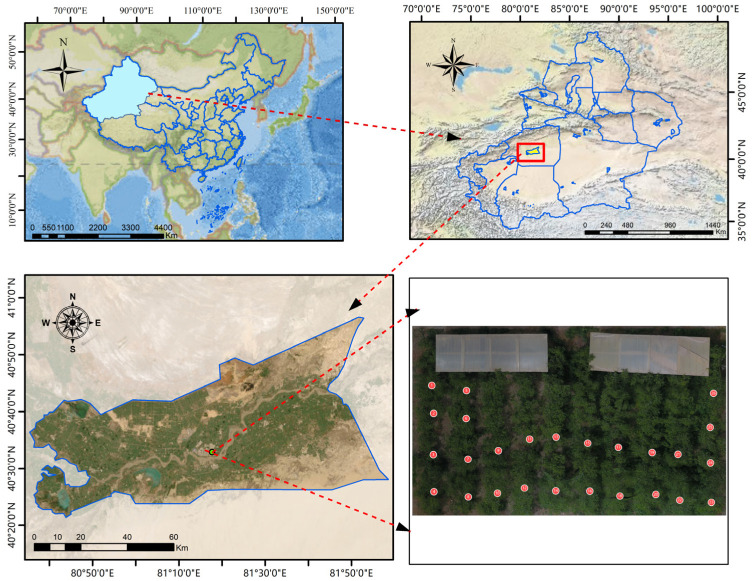
The location of the experimental field is at the Experimental Station of Horticulture, Tarim University, Alar City, Xinjiang Uygur Autonomous Region, China (81°29′ E, 40°54′ N), as indicated by the red line, and the red dots show the labeled locations of the leaf sampling points.

Leaf sampling and data collection were conducted on 25 June 2024, between 10:00 AM and 11:00 AM. In the study area (as shown in Figure 1), the trees were cultivated using a rectangular dense planting method with 2 × 1 m spacing. Healthy leaves with uniform color and no visible pest or disease damage were collected from different phenological stages of Jun jujube [28]. A total of 26 trees were sampled, with 20 leaves collected from each tree.

#### 2.2.2. SPAD Measurement

The chlorophyll content of the leaves was measured using a SPAD-502 portable chlorophyll meter (Konica Minolta, Tokyo, Japan) [11]. Following the SPAD-502 user manual and prior studies on jujube leaf chlorophyll distribution, SPAD readings were taken at five positions (top, upper left, lower left, upper right, lower right) while avoiding the central vein area. This protocol ensured spatial uniformity by sampling across the leaf blade to reduce heterogeneity effects in the mesophyll tissue, aligning with the recommended number of measurement points in the literature [12,13]. The average of these five readings was used to represent the leaf’s SPAD value.

**Figure 2:**
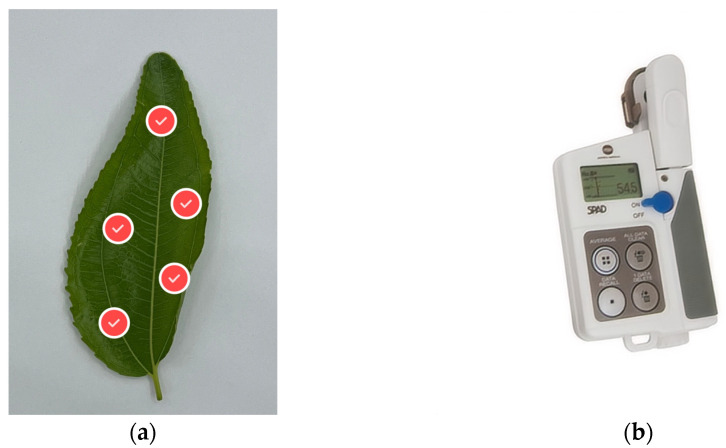
(**a**) SPAD measurement positions on the leaf: top, upper left, lower left, upper right, and lower right, locations of the red dots in the figure, avoiding the central vein; (**b**) SPAD-502 (Konica Minolta, Tokyo, Japan) portable chlorophyll meter used for data collection.

After SPAD measurement, the leaves were promptly placed in a collection box for labeling, storage, and subsequent imaging (as shown in Figure 2).

#### 2.2.3. Image Data Acquisition

**Figure 3:**
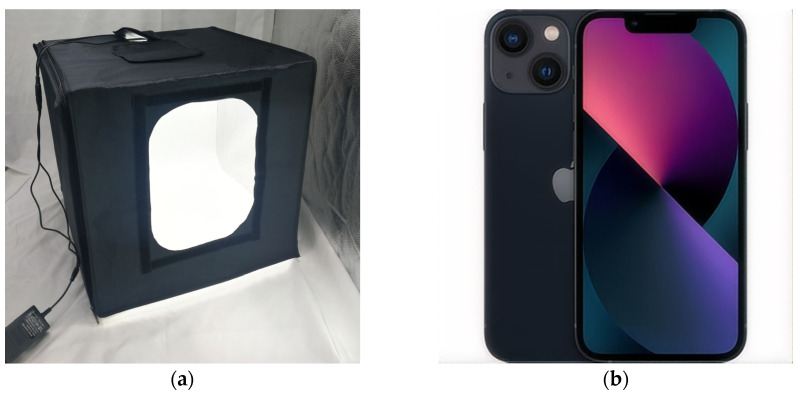
(**a**) Light box setup for leaf image capture; (**b**) iPhone 13 (Apple Inc., Cupertino, CA, USA) smartphone used for imaging.

After collection, the leaves were immediately placed in a prepared photo light box, as shown in Figure 3a. The light box was equipped with two downward-facing LED strips at the top, and its interior was lined with white reflective material on five sides. White A4 paper served as the background on the bottom surface, providing contrast for the leaf placement. Images were captured using a smartphone mounted on a tripod, positioned directly above the leaves at a consistent distance of 30 cm from the lens to the leaf surface.

The light box was designed to eliminate interference from ambient light variations on leaf RGB color features, ensuring data consistency. By standardizing illumination conditions (e.g., fixed LED intensity and color temperature), this study precisely analyzed the correlation between color features and SPAD values, establishing a reliable baseline for model development. For real-world deployment, future work will incorporate color calibration panels or image contrast optimization algorithms [29] to enhance the robustness under natural lighting.

The smartphone used was an iPhone 13 (Figure 3b) equipped with a 12-megapixel rear main camera featuring an ƒ/1.6 aperture, sensor-shift optical image stabilization, and a 5.10 mm focal length. The flash was turned off and the camera settings included an ISO sensitivity of 50, with automatic exposure and shutter adjustments. The images were captured at a resolution of 2268 × 4032 pixels and saved in JPG format.

### 2.3. Image Preprocessing

The leaf images captured in the light box were preprocessed using Python 3.9.19 and OpenCV 4.10.0. First, the images were converted from the RGB color space to the HSV color space. The conversion to the HSV color space was primarily applied for background segmentation. Since the Hue (H) and Saturation (S) channels in HSV are less sensitive to illumination variations, they enabled the robust separation of the leaf region from the white background (high Value, low Saturation). After segmentation, the color features were calculated from the original RGB channels to ensure the direct representation of the leaf’s intrinsic color properties. A color threshold was then applied to remove the background and extract the leaf area. Next, a contour detection method was used to identify the largest contour in the image, which was selected as the region of interest (ROI).

**Figure 4:**
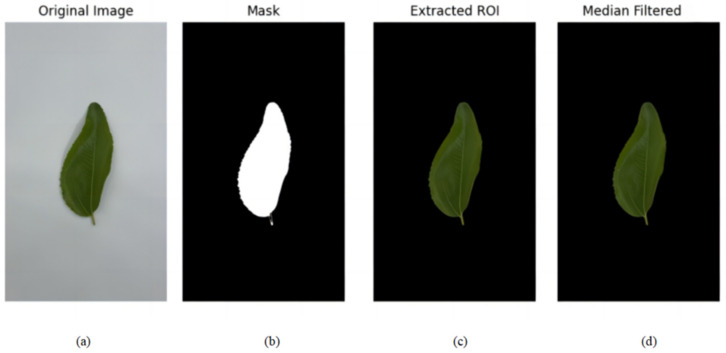
(**a**) Original leaf image; (**b**) background segmentation; (**c**) extracted leaf ROI; (**d**) median filtered leaf image.

A median filter was applied to the ROI to suppress noise and achieve image smoothing, as shown in Figure 4. Finally, the average values of the red, green, and blue color channels in the filtered image were calculated to extract the color features, which were then used for further analysis. This preprocessing workflow effectively eliminated background interference, accurately extracted the leaf region, and generated precise color features for subsequent research.

### 2.4. Selection of Color Features

In this study, a comprehensive analysis of color features was conducted to extract a series of multidimensional features that described the color characteristics of the images [30,31]. As shown in Table 1, these features included the original color channel values R, G, and B, as well as their differences (R − G, R − B, G − B), ratios (R/G, R/B, G/B), and more complex algebraic expressions, such as (G − R)/(G + R), (G + B − R)/(2R), (G + B − R)/(2G), (G + B − R)/(2B), (R − G − B)/(R + B), (R − G − B)/(R + G), (R − G − B)/(G + B), (B − G − R)/(R + B), (B − G − R)/(B + G), (B − G − R)/(G + R), (2G − R − B)/(2G + R + B), (G − B)/(G + B), and (G − B)B/(R + G).

Additionally, normalized color features, denoted as r, g, b, represented the normalized R, G, and B values, along with the differences in these normalized features (r − g, r − b, g − b). These color features comprehensively reflect the distribution, contrast, and correlation of colors in the image, providing valuable data for subsequent image processing and analysis [32,33].

### 2.5. Feature Selection via Correlation and Principal Component Analysis

The correlation coefficient method is a commonly used approach in image processing and data analysis to evaluate the strength of linear relationships between variables. In this study, Pearson correlation analysis was conducted using the Origin 2021 software to assess the linear correlation between color features and SPAD values. Based on the results, color features that showed a high correlation with SPAD values were selected. These features served as key indicators for predicting the chlorophyll content in jujube tree leaves.

To address multicollinearity and reduce dimensionality, principal component analysis (PCA) was applied to the selected features, which further reduced the computational complexity while retaining the core information.

The transformed principal components were used as input variables, with SPAD values as the output variable, to construct an inversion model for estimating the chlorophyll content in jujube tree leaves. The image data were input into the model to predict the SPAD values. Subsequently, the predicted SPAD values were compared with the actually measured ones to determine the accuracy and reliability of the model, see Section 2.5.6 Model Training and Validation.

#### 2.5.1. Support Vector Regression (SVR)

Support Vector Regression (SVR) is a machine learning algorithm used for regression analysis, based on the Support Vector Machine (SVM) framework. It aims to find an optimal hyperplane that minimizes prediction error and can handle nonlinear data. SVR uses a kernel function to map the data into a higher dimensional space, enabling it to accommodate complex data structures.

Support Vector Regression (SVR) constructs a hyperplane that minimizes the distance of all sample points to this hyperplane while allowing a tolerance margin (controlled by parameter ε). The optimization objective is shown in Equation (1):

(1) $$\min_{\omega ,b}\frac{1}{2}{\left\|\omega \right\|}^{2}+C\sum_{i=1}^{n}\left(\xi_{i}+\xi_{i}^{*}\right)$$

Subject to Equation (2):

(2) $$\begin{array}{l} y_{i}-(\omega \cdot \phi (x_{i})-b)\leq \varepsilon +\xi_{i},\\ (\omega \cdot \phi (x_{i})+b-y_{i})\leq \varepsilon +\xi_{i}^{*},\\ \xi_{i},\xi_{i}^{*}\geq 0,i=1,2,\ldots ,n. \end{array}$$

Here, ω is the weight vector, b is the bias term, $\phi (x_{i})$ denotes the kernel mapping, C is the penalty parameter, and $\xi_{i},\xi_{i}^{*}$ are slack variables. A linear kernel $\left(\phi (x_{i})=x_{i}\right)$ was adopted in this study.

The SVR model maps input data to a high-dimensional space via a kernel function and constructs an optimal hyperplane in this space. Support vectors (i.e., samples closest to the hyperplane) determine the model complexity, while the penalty parameter C balances training error and generalization capability. In regression tasks, the ε-band around the hyperplane allows for deviations between predicted and true values, enhancing robustness to noise [25].

#### 2.5.2. Relevance Vector Machine (RVM)

The Relevance Vector Machine (RVM) is a model based on Bayesian inference, similar to the Support Vector Machine (SVM), and is used for both regression and classification tasks. RVM simplifies the model and reduces computational complexity by retaining only those sample points that have a significant impact on the model’s predictions, known as relevance vectors. Compared to SVM, the RVM model is sparser, provides probabilistic predictions, and automatically determines hyperparameters by maximizing the marginal likelihood, without requiring the manual adjustment of penalty parameters.

In experiments, the RVM model uses a linear kernel function, focusing on identifying relevance vectors during the training process to minimize prediction error and build an accurate prediction model.

The mathematical formulation of RVM can be divided into two parts: one is the parameter update formula, and the other is the prediction formula. Below is an overview of the mathematical formulas for RVM regression:

Model Parameter Update Formula: RVM uses a Bayesian framework, where its parameters θ (including those for the Gaussian kernel σ2 and ρ) are estimated by maximizing the likelihood function. The likelihood function includes the predicted values of the model and the true values from the data. The RVM likelihood function can be approximated using Kullback–Leibler (KL) divergence, as shown in Equation (3):

(3) $$L(\theta |X,Y)=\prod_{i=1}^{n}p(y_{i}|x_{i},\theta )$$

In this context, X represents the input data, Y represents the output data, and *n* denotes the number of data points. To find the maximum likelihood estimate of the parameters θ, the negative log-likelihood of the likelihood function needs to be minimized, as shown in Equation (4):

(4) $$-\log L(\theta |x,y)=\sum_{i=1}^{n}\log p(y_{i}|x_{i},\theta )$$

Because $p(y_{i}|x_{i},\theta )$ is typically difficult to compute directly, RVM uses a Gaussian process as its prior distribution and approximates the likelihood function through Kullback–Leibler (KL) divergence. This leads to a method known as Empirical Bayes inference, where the model parameters θ are treated as random variables with known prior distributions, but unknown posterior distributions.

Once the model parameters θ are estimated, RVM can predict the output for new data points $x^{*}$ using the following Equation (5):

(5) $$p(y^{*}|x^{*},X,Y,\theta )=\int p(y^{*}|x^{*},\theta )p(\theta |X,Y)d\theta$$

The term p(θ|X,Y) is typically difficult to integrate directly. To overcome this, RVM employs the Empirical Bayes inference method. This approach estimates the model parameters θ by maximizing the log-likelihood function, which approximates the posterior distribution $p(y^{*}|x^{*},X,Y,\theta )$ of the parameters [26].

#### 2.5.3. CNN Model

Convolutional Neural Networks (CNNs) are a class of deep learning algorithms inspired by biological visual perception mechanisms, and they are particularly well suited for processing images and other data with a grid-like structure. The core advantage of CNNs is their ability to automatically extract features from input data through convolutional operations. By stacking multiple layers, CNNs can learn complex features and patterns. In this study, a CNN model is used for data prediction, and the model structure mainly includes an input layer, a convolutional layer, a pooling layer, a fully connected layer, and an output layer.

**Figure 5:**
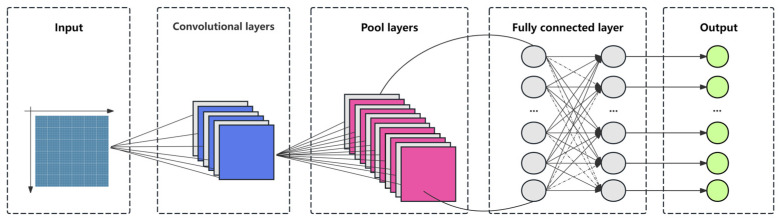
Structure of the Convolutional Neural Network (CNN) model.

In the model (Figure 5), the convolutional layer is responsible for extracting spatial features from the input data, which manifest as local patterns. By designing convolutional kernels of different sizes, the model is able to capture features at varying levels of granularity. Additionally, the pooling layer is an important component of the CNN model. It reduces the spatial dimensions of the feature maps to decrease the number of computational parameters while retaining the most important feature information. This helps prevent overfitting and enhances the model’s ability to generalize. The Adam optimizer is used, along with a learning rate decay strategy, to optimize the model’s convergence. With this design, the model learns hierarchical features, from those that are simple to complex, which ultimately leads to accurate predictions [34].

#### 2.5.4. CNN-SVR Model

**Figure 6:**
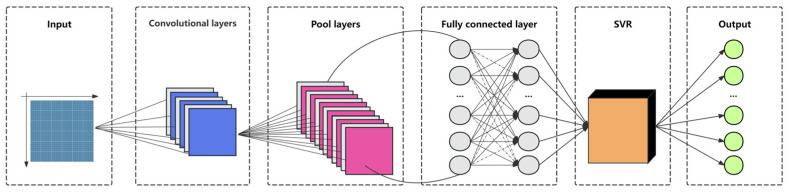
CNN-SVR model structure.

The CNN-SVR model is a prediction model that combines Convolutional Neural Network (CNN) and Support Vector Regression (SVR) to improve the prediction accuracy of small sample data [27]. As shown in Figure 6, the model first uses CNN to automatically learn the deep features of the data. Through the design of convolutional layers, pooling layers, and fully connected layers, it extracts features that are highly relevant to the prediction target. These features are then input into the SVR model for regression prediction. SVR transforms the samples into a high-dimensional feature space through nonlinear mapping, and a linear function is used to fit the samples in that space. The loss is defined as the combined distance of all samples from the linear function, and the parameters of the linear function are determined by minimizing the loss. This approach combines CNN’s powerful feature extraction capabilities with SVR’s efficiency in regression tasks, making it particularly suitable for handling small sample data, reducing the overall training difficulty, and improving prediction accuracy.

#### 2.5.5. CNN-RVM Model

The CNN-RVM model, which combines Convolutional Neural Networks (CNNs) and Relevance Vector Machines (RVMs), is an efficient and powerful hybrid prediction algorithm [35]. This model takes full advantage of CNN’s exceptional feature extraction capability. Through its unique convolutional and pooling layers, CNN automatically learns hierarchical feature representations from raw data, capturing key information crucial for subsequent prediction tasks. At the same time, RVM is known for its high precision in regression prediction and the sparsity of its model. It utilizes the features extracted by CNN to build an accurate and highly generalizable prediction model.

**Figure 7:**
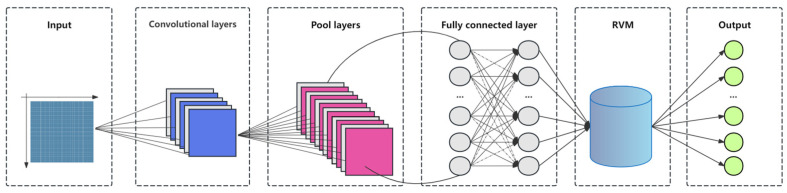
CNN-RVM model structure.

In the overall workflow (Figure 7), CNN first performs deep feature extraction on the input data, transforming the raw data into highly informative high-level feature representations. These features are then passed to RVM for training, where RVM selects the “relevant vectors” that are most critical for prediction, based on its probabilistic model and sparsity principle, thereby constructing a concise and efficient prediction model. During the prediction phase, the model can accurately predict new data points. The CNN-RVM model is particularly suited for handling high-dimensional data with complex internal structures and patterns, such as time series prediction and image analysis tasks. In these domains, the model not only provides precise predictions but also maintains good model interpretability and computational efficiency.

#### 2.5.6. Model Training and Validation

All experiments are conducted on a laptop with an Intel Core i7-10875H CPU (8 cores, 16 threads, 2.30 GHz base frequency), 16 GB DDR4 RAM (3200 MHz), and a 1 TB NVMe SSD. Due to hardware limitations, all models are trained on a CPU without GPU acceleration. The Intel Core i7-10875H processor is designed and manufactured by Intel Corporation, headquartered in Santa Clara, California, U.S.A. This processor is part of its 10th generation of intelligent Core mobile processor family.

The software environment is based on MATLAB R2023a, utilizing the following toolboxes:

Deep Learning Toolbox™: For CNN architecture design and training.

Statistics and Machine Learning Toolbox™: For SVR and RVM implementation.

MathWorks is a global developer and provider of MATLAB and its toolboxes, headquartered in Nettiek, Massachusetts. The latest version of MATLAB R2023a was released in 2023, and the Deep Learning Toolbox™ and Statistics and Machine Learning Toolbox™ is an official MathWorks extension module.

During the model construction process, the hyperparameter optimization and training strategies for each model are carefully designed. For SVR and RVM models, linear, polynomial, and RBF kernel functions are compared by grid search, and the linear kernel is finally selected to achieve a balance between efficiency and generalization, while Bayesian optimization is used to determine the penalty coefficient C in the range of 0.001 to 100. The CNN model adopts a shallow network architecture, with a 2-layer convolution and 16–32 filters in each layer to prevent overfitting, and the training period is dynamically controlled by the early-stop method (with a patience value set to 10). The training cycle is dynamically controlled by the early stopping method (with a patience value of 10), and the initial learning rate is set to 0.01, which is adjusted by the cosine annealing strategy. In the hybrid model, the CNN and SVR/RVM are trained in stages to improve the overall model performance.

In model evaluation, accurately assessing the performance of a predictive model is crucial to ensure its effectiveness and robustness. This process plays a decisive role in determining the precision of model predictions. In this context, a series of specialized performance evaluation metrics are widely used, including the coefficient of determination (R^2^) for both the training and prediction datasets, as well as the Root Mean Squared Error (RMSE) for both the training and prediction datasets in Equation (6). A high R^2^ value indicates that the model has a high predictive power and can effectively explain the variation in the data, while a low RMSE value indicates that the model exhibits high stability and accuracy during the prediction process.

Root Mean Squared Error (RMSE):

(6) $$RMSE=\sqrt{\frac{1}{n}\sum_{i=1}^{n}{\left(y_{i}-{\hat{y}}_{i}\right)}^{2}}$$

Coefficient of determination(R^2^):

(7) $$R^{2}=1-\frac{\sum_{i=1}^{n}{\left(y_{i}-{\hat{y}}_{i}\right)}^{2}}{\sum_{i=1}^{n}{\left(y_{i}-\bar{y}\right)}^{2}}$$

The formula defines *n* as the number of samples, $y_{i}$ as the actual observation value for the i sample, ${\hat{y}}_{i}$ as the predicted value for the i sample, and $\bar{y}$ as the mean of all actual observation values in Equation (7).

To further validate the model generalizability, we implement 5-fold cross-validation. The dataset is randomly split into 5 mutually exclusive subsets. In each iteration, 4 subsets are used for training, and the remaining subset for validation. The mean R^2^, RMSE, and their standard deviations are calculated over 5 repetitions.

## 3. Results

### 3.1. Measurement Results

**Figure 8:**
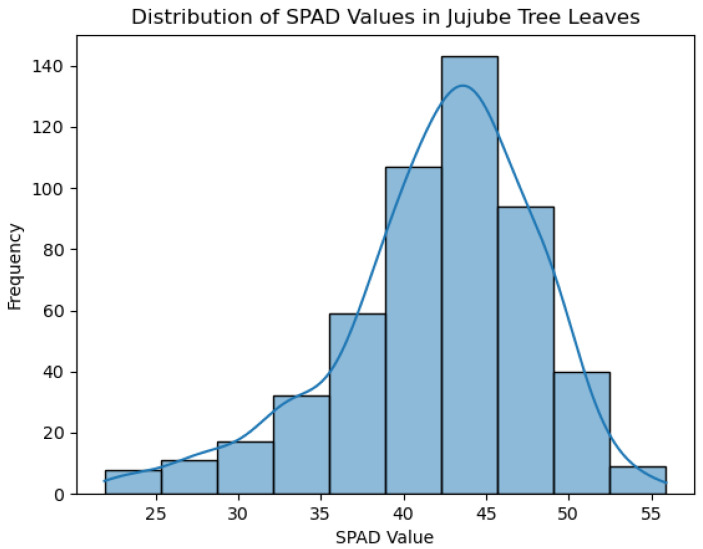
Distribution of SPAD values for 520 leaf samples.

As shown in Figure 8, the distribution of chlorophyll SPAD values is presented for 520 jujube leaf samples. The minimum value is 21.90, the maximum value is 55.90, the average value is 42.05, the standard deviation is 5.99, and the median is 43.05.

### 3.2. Correlation Analysis Between Color Features and Chlorophyll Content

**Figure 9:**
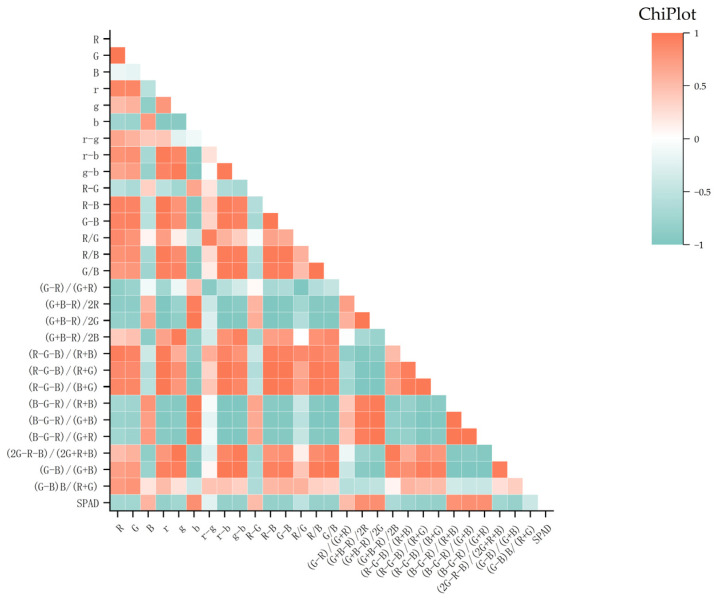
Pearson correlation analysis between color features and SPAD values.

In this study, the Origin 2021 software was used to extract color features from the visible light images of 520 different jujube tree leaves, and a correlation analysis was performed between the color features and the chlorophyll content. Figure 9 shows the results of the color features significantly correlated with the chlorophyll content of the jujube tree leaves. The analysis revealed a significant positive correlation between the SPAD value and the color feature (G + B − R)/2G, with a correlation coefficient of 0.84. Meanwhile, the SPAD value showed a significant negative correlation with the color feature R − B, with a correlation coefficient of −0.84. In comparison, the correlation with the normalized color feature r − g was weaker, with a correlation coefficient of −0.23.

Based on the analysis results in Figure 9, most color features exhibited a strong correlation with chlorophyll content, with correlation coefficients generally exceeding 0.7. Accordingly, 21 color features with a strong correlation to chlorophyll content were selected, including R, G, r, g, b, r − b, g − b, R − B, G − B, R/B, G/B, (G + B − R)/(2R), (G + B − R)/(2G), (R − G − B)/(R + B), (R − G − B)/(R + G), (R − G − B)/(G + B), (B − G − R)/(R + B), (B − G − R)/(B + G), (B − G − R)/(G + R), (2G − R − B)/(2G + R + B), and (G − B)/(G + B). These features could be used to support the construction of prediction models.

In order to eliminate the multicollinearity among color features, this study used principal component analysis (PCA) to downscale the 21 initial features. The cumulative variance contribution ratio of the first five principal components (PC1 to PC5) was 99.99%, which fully retained the effective information of the original data (Table 2). Based on the PCA score matrix, PC1 to PC5 were extracted as new input features for model training and validation.

### 3.3. The Best Model for Predicting SPAD

In the subsequent experiment, PC1 to PC5 were used as input features for model training and validation. The training and validation data were split in an 80:20 ratio. Various models were applied to predict SPAD content and were compared. The models used included Support Vector Regression (SVR), Relevance Vector Machine (RVM), Convolutional Neural Network (CNN), CNN combined with SVR (CNN-SVR), and CNN combined with RVM (CNN-RVM).

**Figure 10:**
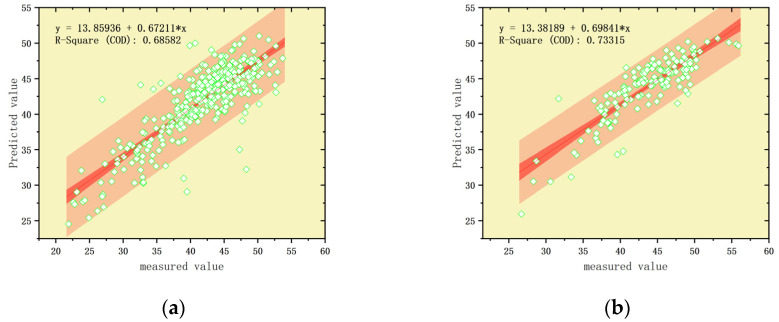

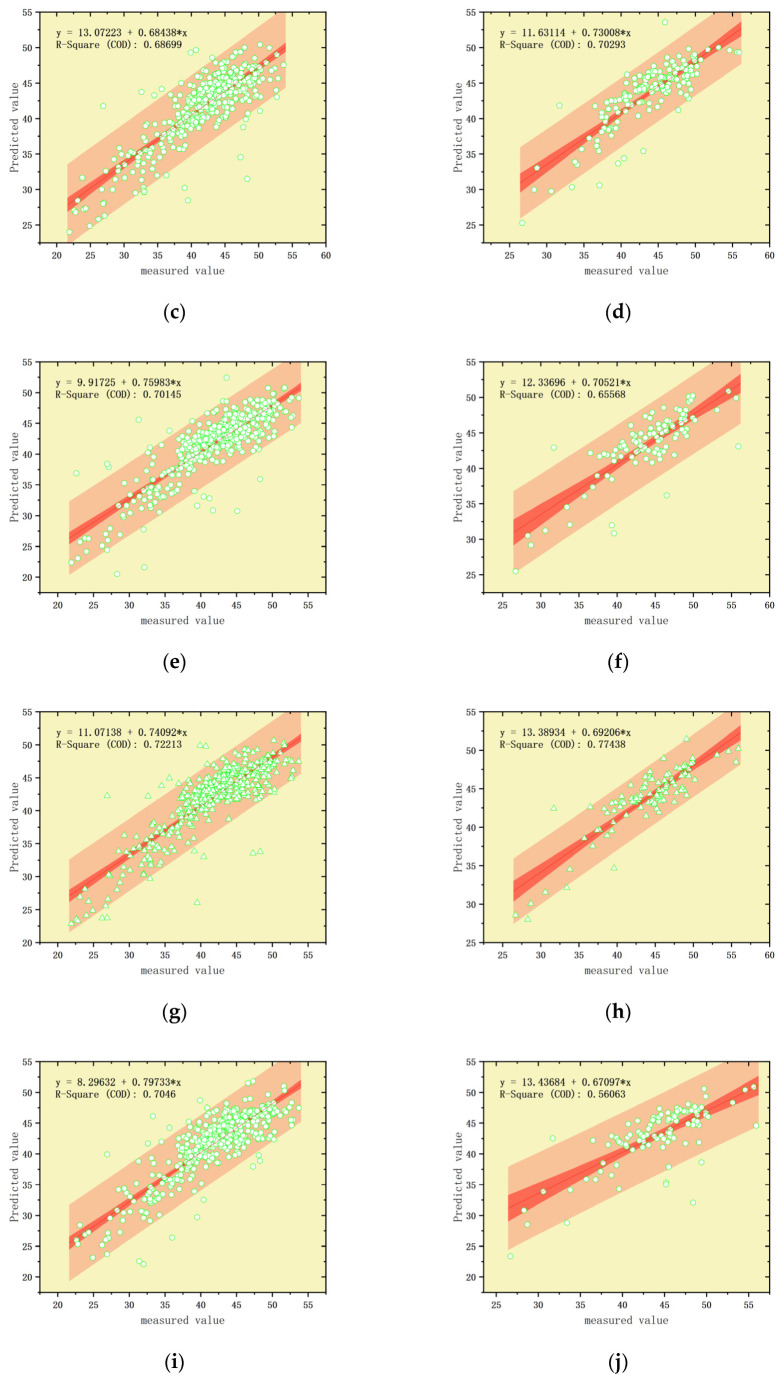
(**a**) Scatter plot of prediction results of SVR model training set; (**b**) scatter plot of prediction results of SVR model validation set; (**c**) scatter plot of prediction results of RVM model training set; (**d**) scatter plot of prediction results of RVM model validation set; (**e**) scatter plot of prediction results of CNN model training set; (**f**) scatter plot of prediction results of CNN model validation set; (**g**) scatter plot of prediction results of CNN-SVR model training set; (**h**) scatter plot of prediction results of CNN-SVR model validation set; (**i**) scatter plot of prediction results of CNN-RVM model training set; (**j**) scatter plot of prediction results of CNN-RVM model validation set.

During the training of the Support Vector Regression (SVR) model, the penalty coefficient C was set to 0.01, and the kernel function parameter γ was set to 150, with a linear kernel function used for training. As shown in Figure 10a, the SVR model exhibited slightly higher error on the training set, with an RMSE of 3.30 and an R^2^ of 68.58%. Compared to CNN-based models, the SVR model showed relatively poorer fitting performance on the training set. However, on the validation set, as depicted in Figure 10b, the SVR model achieved an RMSE of 2.74 and an R^2^ of 73.32%.

The Relevance Vector Machine (RVM) model employed a Gaussian kernel function to handle the nonlinear relationships in the data, with the kernel function parameter γ set to 0.01, which controlled the kernel’s influence range. On the training set, as shown in Figure 10c, the RVM model achieved an RMSE of 3.30 and an R^2^ of 68.69%. Although the RVM model’s performance was slightly weaker, its R^2^ was marginally higher than that of the SVR model, demonstrating competitive capability. However, on the validation set, as shown in Figure 10d, the RVM model achieved an RMSE of 2.70 and an R^2^ of 70.29%. The RVM model exhibited relatively stable performance on the validation set but was slightly inferior to the SVR model.

The Convolutional Neural Network (CNN) was trained for 1000 epochs for the regression task. The network architecture consisted of an input layer, convolutional layers, batch normalization layers, ReLU activation layers, max pooling layers, fully connected layers, and a regression layer. The convolutional layers used a 3 × 1 kernel to generate 16 feature maps, introducing nonlinearity through the ReLU activation function. The max pooling layers had a window size of 2 × 1 and a stride of 2 for dimensionality reduction. The model was trained using the Adam optimization algorithm with an initial learning rate of 0.01, which decayed to 0.1 of its original value every 100 epochs. To prevent overfitting, L2 regularization (with a coefficient of 0.001) and gradient clipping (threshold of 1.0) were employed.

On the training set, as shown in Figure 10e, the CNN model achieved a Root Mean Squared Error (RMSE) of 3.00 and an R^2^ of 70.15%. The model demonstrated a robust performance with low errors, and the R^2^ indicated that it explained most of the variance. On the validation set, as shown in Figure 10f, the CNN model achieved an RMSE of 2.95 and an R^2^ of 65.57%. The consistent performance between the training and validation sets suggested that the CNN model had a good generalization ability.

The hybrid model combining Convolutional Neural Network (CNN) and Support Vector Regression (SVR) consisted of a CNN component with an input layer, convolutional layers, batch normalization layers, ReLU activation layers, max pooling layers, fully connected layers, and a regression layer. After 1000 training epochs, the model was optimized using the Adam algorithm with an initial learning rate of 0.01, which was gradually reduced during training to enhance performance. The features extracted by CNN were subsequently input into an SVR model for further predictions. The SVR used a linear kernel, a penalty coefficient C of 0.01, and a kernel parameter γ = 150.

The CNN-SVR model’s performance was validated on both the training and testing datasets. The results showed that the combined model produced similar results to the standalone CNN model on the training set (Figure 10g), with an RMSE of 3.10 and an R^2^ of 73.46%, indicating no significant improvement from integrating SVR. However, on the validation set Figure 10h, the CNN-SVR model achieved an RMSE of 2.82 and an R^2^ of 77.18%. Compared to the standalone CNN, the CNN-SVR model demonstrated slightly better performance on the validation set, particularly in terms of relative error and goodness of fit, highlighting the added precision provided by the SVR component.

The hybrid model combining Convolutional Neural Network (CNN) and Relevance Vector Machine Regression (RVR) integrated the feature extraction capabilities of CNN with the nonlinear regression strength of RVR. The CNN component consisted of multiple layers: the convolution and pooling operations extracted features from input data, followed by the fully connected layers and a regression layer for prediction. The convolution layers employed 3 × 1 and 2 × 1 kernels, generating 16 and 32 feature maps, respectively, while the max pooling layers reduced feature dimensionality. The CNN was trained using the Adam optimizer with an initial learning rate of 0.01, dynamically adjusted during the maximum 1000 training epochs. After training, features from an intermediate layer (“pool2”) were extracted and fed into the RVR model for regression analysis.

The RVR employd a Gaussian kernel with a gamma parameter of 0.05, leveraging the Relevance Vector Machine for regression tasks. By combining CNN’s feature extraction ability and RVR’s nonlinear regression capacity, the model achieved enhanced predictive performance.

As shown in the results, the CNN-RVM model achieved an RMSE of 3.05 and R^2^ of 70.46% on the training set (Figure 10i), outperforming the previously discussed models, particularly in terms of R^2^, which indicated its stronger explanatory power for the training data. However, on the validation set in Figure 10j, the model’s RMSE increased to 3.50, and R^2^ dropped significantly to 56.06%, reflecting a notable decrease in generalization capability. This sharp decline suggested that the CNN-RVM model suffered from overfitting, excelling on the training data but struggling to generalize to unseen data.

The results, as shown in Table 3, indicated that the CNN-SVR hybrid model demonstrated the best overall performance. On the training set, the CNN-SVR model achieved a Root Mean Squared Error (RMSE) of 3.10 and an R^2^ of 72.21%, closely matching the performance of the standalone CNN model. This suggested that the hybrid model maintained robust fitting capability for the training data. On the validation set, the CNN-SVR model recorded an RMSE of 2.50 and an R^2^ of 77.44%, showing a notable improvement over the CNN model, which had an RMSE of 2.95 and an R^2^ of 65.57%. This highlighted CNN-SVR’s superior performance in terms of goodness-of-fit and error metrics.

Although the SVR model achieved the highest R^2^ of 73.32% on the validation set, its training set performance was relatively poor, with an RMSE of 3.30 and R^2^ of 68.58%, indicating potential underfitting to the training data.

In contrast, the CNN-SVR hybrid model exhibited consistent error metrics across the training and validation sets, reflecting a stronger generalization capability while effectively avoiding the overfitting issues observed in other models, such as CNN-RVM. Consequently, the CNN-SVR hybrid model stood out as the optimal model for predicting SPAD content in this study, providing stable and reliable performance across both datasets.

## 4. Discussion

In this study, the SPAD values of jujube leaves were obtained using a SPAD meter, while corresponding RGB images were captured using a smartphone. The color components of the leaves were extracted, and various color features were calculated. Through correlation analysis, color features that were significantly correlated with SPAD values were identified. In order to eliminate the multicollinearity between the color features, principal component analysis (PCA) was used in this study to downscale these color features. The dimensionality-reduced principal components were used as inputs to prediction models based on the SVR, RVM, CNN, CNN-SVR, and CNN-RVM algorithms. The results were then summarized and analyzed to determine the most effective predictive algorithm. Cross-validation results showed that the CNN-SVR hybrid model exhibited the highest validation R^2^ (77.44%) and the lowest standard deviation (±2.50%), confirming its robustness to changes in data partitioning. Its training stability (±4.10% R^2^ deviation) further highlighted its applicability in small-sample situations. CNN-RVM exhibited a significant performance degradation on the validation set (R^2^ = 56.06%, RMSE = 3.50) and high variability (±4.56%), indicating its sensitivity to the training data distribution. The validation R^2^ values of SVR and RVM were moderate (73.32% and 70.29%, respectively) but with high variability (±4.09% and ± 5.06%), which reinforced the advantages of integrating CNN with SVR.

In this study, differences in the strengths and weaknesses of various models resulted in variations in their generalization abilities and fitting performance. Support Vector Regression (SVR), employing a linear kernel, demonstrated relatively simple implementation and a strong generalization capability on the validation set (R^2^ = 73.32%, RMSE = 2.74). However, it exhibited poor fitting on the training set (R^2^ = 68.58%, RMSE = 3.30), indicating underfitting to the training data. Relevance Vector Regression (RVM), which utilizes a Gaussian kernel and theoretically has the advantage of sparsity, showed moderate performance on the training set (R^2^ = 68.69%, RMSE = 3.30) but underperformed on the validation set (R^2^ = 70.29%). Additionally, its implementation was complex, and its computational cost was high.

A study on soybean chlorophyll inversion [36] demonstrated that the performance of different regression models (e.g., RF, BPNN, SVM) varies across growth stages. For example, the Random Forest model performed best during the full podding stage, achieving an R^2^ of 0.8, while the BPNN model performed better during the flowering stage with an R^2^ of 0.7. This indicates that model selection and crop growth stages significantly influence prediction accuracy.

Convolutional Neural Networks (CNNs) demonstrated excellent feature extraction capabilities through multiple layers of convolution, pooling, and fully connected layers. Although their code implementation was complex and demanded substantial computational resources, it showed consistent performance on both the training set (R^2^ = 70.15%) and validation set (R^2^ = 65.57%), indicating a strong generalization ability. The CNN-SVR hybrid model, combining CNN’s feature extraction and SVR’s regression ability, showed the best performance on the validation set (R^2^ = 77.44%, RMSE = 2.50), highlighting stable generalization and making it the optimal model in this study. In contrast, CNN-RVM, while performing excellently on the training set (R^2^ = 70.46%, RMSE = 3.05), suffered from overfitting and performed poorly on the validation set (R^2^ = 56.06%). Thus, considering model performance and implementation complexity, CNN-SVR struck the best balance between stability, generalization ability, and code implementation difficulty, making it the recommended model for predicting SPAD values.

This study used a combined approach of SPAD testing and RGB image analysis to estimate the SPAD values of jujube leaves. The effectiveness of color indices is rooted in the chlorophyll absorption spectrum of jujube leaves. Chlorophyll-a/b strongly absorbs blue (430–450 nm) and red (640–660 nm) wavelengths while reflecting green (500–580 nm) light. Thus, indices combining green (G) and blue (B) while suppressing red (R) (e.g., (G + B − R)/2G) maximize chlorophyll signals. The numerator (G + B − R) enhances chlorophyll-related reflection, while the denominator (2G) normalizes illumination variations, adhering to the path-length correction principle of the Beer–Lambert law. In contrast, simple ratios (e.g., R/G) are prone to ambient light fluctuations and non-chlorophyll pigments (e.g., anthocyanin under drought stress), reducing their reliability [8].

Similar studies have shown that leaf color features extracted from RGB images can effectively predict SPAD values. Compared to traditional SPAD devices, this method is more cost-effective and convenient. In [37], the SPAD values and color features of tree leaves were analyzed, validating the significant correlation between SPAD values and chlorophyll content, suggesting that RGB image analysis is a reliable alternative method. Similarly, in [38], a study on pomegranate tree leaves using multiple linear regression with G and B values for SPAD prediction (R^2^ = 83.55%) achieved high accuracy, further supporting the broad application of RGB image analysis in agriculture.

Moreover, this study incorporated various machine learning models, such as SVR, RVM, and CNN, combined with leaf color features, for SPAD value prediction. Research in [24] indicated that CNNs, combined with RGB images, perform well in chlorophyll estimation (R^2^ = 82%), particularly in handling nonlinear data, where CNN’s feature extraction ability outperforms traditional regression methods. Therefore, our experimental results align with these previous findings, demonstrating that RGB image-based SPAD value estimation using machine learning not only simplifies the workflow but also improves the prediction accuracy and generalization ability.

## 5. Conclusions

This study proposes a method for SPAD estimation based on smartphone images. In our experimental environment, which involved collecting data from 520 jujube leaf samples in the arid region of Alar, Xinjiang, and using a specific image preprocessing and model training approach, the CNN–SVR hybrid model demonstrated distinct advantages in SPAD prediction. It achieved an R^2^ of 72.21% and an RMSE of 3.10 on the training set, and an R^2^ of 77.44% and an RMSE of 2.50 on the validation set. These results showed that, within the scope of our experiment, the CNN–SVR model had better fitting and generalization capabilities compared to individual models such as SVR, RVM, and CNN. Based on our experimental findings, the CNN–SVR model shows potential for practical application in the jujube orchards in the study area. However, due to the lack of multi-site and multi-temporal testing, as well as real-world variability validation, its broader applicability in different agricultural environments and with different crop types remains to be further investigated. Future research is needed to fully explore its potential in various practical scenarios.

However, there were some limitations in this study, including the limited size of the dataset, the image resolution, and the environmental factors that may have affected feature extraction accuracy. Additionally, the process of selecting color features relied on manual methods. Future work can improve the model’s accuracy and applicability by expanding the dataset, incorporating multispectral or hyperspectral images, optimizing deep learning model structures, and implementing automated feature selection. In terms of the adaptive preprocessing pipeline, the dynamic white balance was achieved by integrating the ambient light sensor of the smartphone, and the white balance parameters were automatically adjusted, effectively reducing the color shift caused by light. Background segmentation using a lightweight U-Net model trained on multi-environment leaf images can remove complex backgrounds in real time and improve image quality. At the same time, the RGB values under different lighting conditions were normalized by embedding a micro color reference card or using an image contrast optimization algorithm for calibration. Robust feature engineering enhanced the stability of features by replacing the original RGB values with illumination invariants that were less sensitive to changes in brightness, such as (G − B)/(G + B) or excess green index (E × G). With CycleGAN, multiple lighting training was used to enrich the dataset by synthesizing data from different lighting conditions (e.g., cloudy days, direct sunlight), which significantly improved the versatility of the model. At the device optimization level, the CNN–SVR model was integrated into mobile phone applications, which reduced the inference time of each image on smartphones and improved the efficiency of the model running on mobile devices. In the field validation program, cross-site tests were carried out to validate the model in jujube orchards in different arid regions to evaluate its geographical versatility. In addition, intuitive mobile apps with an offline mode that are farmer-centric and provide instant SPAD feedback and agronomic recommendations (e.g., irrigation planning) should be developed to drive the application of the research findings to real-world agricultural production.

Looking ahead, the future development directions of this study include expanding the dataset and sample range to enhance the model’s generalization ability and applicability across different plant species; optimizing deep learning models by introducing updated network structures or automated hyperparameter tuning techniques; adopting multispectral or hyperspectral imaging to obtain more image information, thereby improving prediction accuracy; and developing real-time monitoring systems to apply the model in practical agricultural production for more convenient SPAD estimation. Moreover, the limitations of this study, such as the small sample size and the restricted plant species, as well as the limited resolution of smartphone images and the potential impact of environmental conditions, may affect the feature extraction accuracy. Additionally, the reliance on manual methods for color feature selection can be addressed in the future by automating the feature selection process to improve the model’s reliability and precision.

## Figures and Tables

**Table 1:** Selected color features for image analysis.

Color Space	Number	Color Characteristics
RGB	22	R, G, B, R − G, R − B, G − B, R/G, R/B, G/B, (G − R)/(G + R), (G + B − R)/(2R), (G + B − R)/(2G), (G + B − R)/(2B), (R − G − B)/(R + B), (R − G − B)/(R + G), (R − G − B)/(G + B), (B − G − R)/(R + B), (B − G − R)/(B + G), (B − G − R)/(G + R), (2G − R − B)/(2G + R + B), (G − B)/(G + B), (G − B)B/(R + G)
Normalized RGB	6	r, g, b, r − g, r − b, g − b

**Table 2:** Principal component variance contribution.

Principal Component	Variance Contribution	Cumulative Variance Contribution
PC1	92.03831%	92.03831%
PC2	6.68462%	98.72294%
PC3	0.82799%	99.55093%
PC4	0.41319%	99.96413%
PC5	0.02890%	99.99303%
PC6	0.00366%	99.99669%
PC7	0.00209%	99.99878%
PC8	0.00103%	99.99981%
PC9	0.00007%	99.99989%
PC10	0.00007%	99.99996%
PC11	0.00002%	99.99997%
PC12	0.00001%	99.99998%
PC13	0.00001%	99.99999%
PC14	0.00000%	99.99999%
PC15	0.00000%	100.00000%
PC16	0.00000%	100.00000%
PC17	0.00000%	100.00000%
PC18	0.00000%	100.00000%
PC19	0.00000%	100.00000%
PC20	0.00000%	100.00000%
PC21	0.00000%	100.00000%

**Table 3:** Performance of chlorophyll prediction models.

Model	Training Set				Validation Set			
	R^2^	Std	RMSE	Std	R^2^	Std	RMSE	Std
SVR	68.58%	6.04%	3.30	0.034	73.32%	4.09%	2.74	0.039
RVM	68.69%	5.69%	3.30	0.032	70.29%	5.06%	2.70	0.037
CNN	70.15%	4.59%	3.00	0.035	65.57%	3.82%	2.95	0.041
CNN-SVR	72.21%	4.10%	3.10	0.027	77.44%	2.50%	2.50	0.035
CNN-RVM	70.46%	4.95%	3.05	0.041	56.06%	4.56%	3.50	0.120

## Data Availability

The data involved in this study can be obtained by contacting the authors.
